# Wnt signaling pathway-related gene PRICKLE1 is a prognostic biomarker for esophageal squamous cell carcinoma

**DOI:** 10.3389/fonc.2022.1014902

**Published:** 2023-02-13

**Authors:** Jinxian He, Gaofeng Liang, Hui Tian, Yiqing Wang, Li Yu, Wang Lv, Jian Hu, Weiyu Shen

**Affiliations:** ^1^ Department of Thoracic Surgery, The First Affiliated Hospital, Zhejiang University School of Medicine, Hangzhou, Zhejiang, China; ^2^ Department of Thoracic Surgery, Ningbo Medical Center Lihuili Hospital, Ningbo University, Ningbo, Zhejiang, China

**Keywords:** esophageal squamous cell carcinoma, PRICKLE1, Wnt signaling pathway, overall survival, proliferation, cell migration, apoptosis

## Abstract

Esophageal squamous cell carcinoma (ESCC) has become a major health risk to human health. Although significant clinical progress has been made in the treatment of ESCC, the prognosis of patients still needs to be improved. Therefore, it is important to screen effective molecular indicators for the prognosis of ESCC. In this study, the intersection of up-regulated genes, down-regulated genes, and Wnt signaling pathway-related genes in ESCC was taken, and 47 overlapping genes were found. PRICKLE1 was determined to be an independent prognostic factor in ESCC based on univariate and multifactorial COX risk regression models. Kaplan-Meier survival curves showed that patients in the PRICKLE1 high expression group had significantly better overall survival. In addition, we performed various experiments to examine the effects of PRICKLE1 overexpression on proliferation, migration, and apoptosis of ESCC cells. The experimental results showed that the PRICKLE1-OE group had reduced cell viability, significantly lower migration ability and significantly higher apoptosis rate compared to the NC group.Therefore, we hypothesized that high PRICKLE1 expression could be used to predict the survival rate of ESCC patients, which could be used as an independent prognostic indicator for ESCC patients and provide potential applications for ESCC clinical treatment.

## 1 Introduction

The incidence of esophageal cancer has shown a significantly increasing trend. The main manifestations of esophageal cancer are damage to the esophageal wall and narrowing of the esophageal lumen, and patients often have symptoms such as difficulty in swallowing, dryness, and tightness in the throat (1). Esophageal cancer is very easy to invade and infiltrate and grow, and the symptoms are insidious, so the first diagnosis is often a patient in the middle or late stage, so the annual survival rate is not high (2). In 2020, the global cancer incidence of esophageal cancer is reported to be 600,000, ranking eighth, and the number of global deaths from esophageal cancer is reported to be 540,000, ranking sixth, making esophageal cancer a major health hazard to human beings (3). The causes of esophageal cancer are still unclear, and it is believed that the occurrence of the disease is influenced by a combination of factors, such as genetics, lifestyle habits, dietary structure, alcoholism and smoking, and environmental characteristics, which are all related to the occurrence of esophageal cancer (4, 5). The histological typing of esophageal cancer is mainly ESCC andesophageal adenocarcinoma, among which, the pathological type of esophageal cancer in China is mainly ESCC, which is more common than esophageal adenocarcinoma (6). Although significant clinical progress has been made in the treatment of ESCC in recent years, the prognosis of patients still needs to be improved. Studies have shown that the occurrence and progression of ESCC involve multi-gene and multi-pathway alterations (7). Therefore, it is important to screen effective molecular indicators for population screening and prognosis determination of ESCC, which can help prolong the survival time of patients.

The Wnt signaling pathway is one of the five major cell signaling pathways and consists of a series of proteins encoded by oncogenes and oncogenes, which are regulated by several key proteins that are interconnected and mutually regulated (8). The Wnt signaling pathway is evolutionarily conserved and is involved in regulating cell growth and development, differentiation, proliferation, migration, and other growth processes (9). Abnormalities in the Wnt signaling pathway activation may lead to malignant transformation of cells and tumor development, invasion, and metastasis, and are closely related to poor patient prognosis (10). In recent years, this pathway has been a hot topic of research in cancers such as gastric, hepatocellular, colorectal, breast, and pancreatic cancers, and certain results have been achieved (11, 12). This study aimed to identify Wnt signaling pathway genes associated with ESCC prognosis, construct a Nomogram model, analyze single-gene prognosis, and experimentally validate them to improve patients’ quality of life.

## 2 Methodology

### 2.1 Data sources

RNA-seq data of ESCC and its clinical information were downloaded from The Cancer Genome Atlas database, containing ESCC samples (N=82) and paracancerous tissue samples (N=11), clinical information included data on the age, race, clinical stage, and overall survival (OS) of the sample, with OS defined as the time from any cause or last follow-up diagnosis to death.Wnt signaling pathway-related genes (N=167) were downloaded from the MSigDB database (https://www.gsea-msigdb.org/gsea/msigdb/index.jsp).

### 2.2 Differential expression analysis

Using P<0.05 and |Log2FC|>1 as the screening threshold for differentially expressed genes (DEGs), differential analysis of ESCC cancer and paraneoplastic tissues was performed using the R software “limma” package. Venn diagrams were used to intersect the DEGs and Wnt signaling pathway-related genes to obtain the differentially expressed Wnt signaling pathway-related genes in ESCC.

### 2.3 Identification of prognosis-related genes

One-way COX regression analysis was performed on DEGs related to the Wnt signaling pathway, genes related to prognosis were screened using the Log-rank test, and forestplot was plotted using the R package “forestplot”.

### 2.4 Nomogram construction

Independent influences on patient prognosis were determined based on univariate and multifactorial COX risk regression models. Based on the independent influences on prognosis, a Nomogram was constructed using the R package “rms” and a line graph was drawn.

### 2.5 Single-gene prognostic analysis

The overall survival of patients in the single gene high and low expression groups was analyzed by the R package Kaplan-Meier and Log-rank test. ROC curves were used to assess the predictive performance of prognostic genes for 1, 2, and 3-year survival of patients.

### 2.6 Cell culture and processing

The human esophageal cancer KYSE-450 cell line and KYSE-410 cell line and human normal esophageal squamous epithelial cell Het-1A was inoculated in RPMI 1640 culture medium (containing 10% fetal bovine serum, penicillin 100 U/mL, and streptomycin 100 μg/mL) and cultured at 37°C, 5% CO_2_, and saturated humidity, and the culture medium was changed once in 2 d. When the cell growth density reached about 90%, the cells were passaged at a ratio of 1:2. Cells in the logarithmic growth phase were taken for experiments.

Cells were transfected with lentiviral overexpression vector or empty vector using Lipofectamine 2000 reagent. The cells were transfected with the PRICKLE1-OE group and control group respectively, and the transfected cells were incubated at 37°C for 24 h. Refer to the kit instructions for the specific transfection operation.

### 2.7 Real-time quantitative polymerase chain reaction

Total RNA was extracted using the TRIzol kit (Invitrogen, USA). cDNA was synthesized using the reverse transcription kit. qPCR was performed using the SYBR Premix Ex Taq kit (TaKaRa, Japan). GAPDH was used as an internal reference, and the primer sequences were as in **Table 1**. follow the kit instructions. The experiment was repeated 3 times.

**Table 1:** qPCR primer sequence.

Gene	Forward 5’-3’	Reverse 5’-3’
PRICKLE1	TTTGCTTGCTTACCAGAGGAAA	ACTGGCAATACCGTACCTCAT
GAPDH	CTGGGCTACACTGAGCACC	AAGTGGTCGTTGAGGGCAATG

### 2.8 Immunoblotting assay

Cells were lysed using cell lysis buffer. total protein concentration was determined by BCA method. Proteins were separated using SDS-PAGE electrophoresis and transferred to PVDF membranes. After sealing with 5% skim milk, the membranes were incubated with primary antibodies overnight. The primary antibody consisted of Anti-β Catenin (ab32572, abcam). Membranes were washed 3 times with TBST and incubated with horseradish peroxidase-coupled secondary antibodies at room temperature. Target bands were detected using ECL. GAPDH (ab8245, abcam) was used as an internal control. Repeat the experiment 3 times.

### 2.9 CCK-8

Esophageal cancer cells overexpressing PRICKLE1 were digested and inoculated into 96-well plates, and the cell density was adjusted to 1*10^4^ cells per well using culture medium, with 3 replicate wells per group. Add 10 μl CCK-8 solution to each well, incubate in the incubator for 2 h, and detect the absorbance at 450 nm using an enzyme marker. The percentage of cell viability was obtained.

### 2.10 Cell migration

A marker pen was used to draw horizontal lines evenly across the back of the 6-well plate, approximately every 0.5~1 cm, across the wells, with at least 5 lines across each well. About 5×10^5^ cells were added to the wells, and the next day the marker was used with the tip of the gun compared to a straightedge and scratched as far down as possible to the horizontal line behind. Wash the cells with PBS 3 times, remove the scratched down cells, add serum-free medium; put into 37°C, 5% CO_2_ incubator and incubate; take samples according to 0h and 24h, observe the migration of cells at specific locations with inverted microscope and take pictures.

### 2.11 Apoptosis

Collect the cells, resuspend 1×10^5^ cells in 200μL Binding Buffer, and add 4μL 0.5 mg/mL PI and 2μL Annexin V-FITC solution. Incubate at room temperature for 15 min at avoidance of light, and perform fluorescence detection by flow cytometry.

### 2.12 Statistical processing

GraphPad Prism 6 was used for data processing. The measurement data were expressed as mean ± standard deviation, and the data were compared between groups using a t-tset. all experiments were repeated three times. p<0.05 was considered to be a significant difference.

## 3 Results

### 3.1 Differential expression analysis

**Figure 1:**
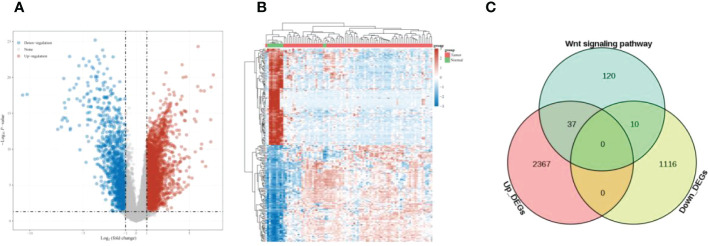
Differential expression analysis **(A)** Volcano plot showing DEGs of ESCC; **(B)** Heat map showing the distribution of DEGs of ESCC; **(C)** Venn diagram, overlapping genes were obtained.

Differential analysis of ESCC samples and paracancer tissue samples yielded 3451 up-regulated genes and 761 down-regulated genes (**Figures 1A, B**). The intersection of up-regulated genes, down-regulated genes, and Wnt signaling pathway-related genes in ESCC was taken, and 47 overlapping genes were found (**Figure 1C**).

### 3.2 Identification of prognosis-related genes

**Figure 2:**
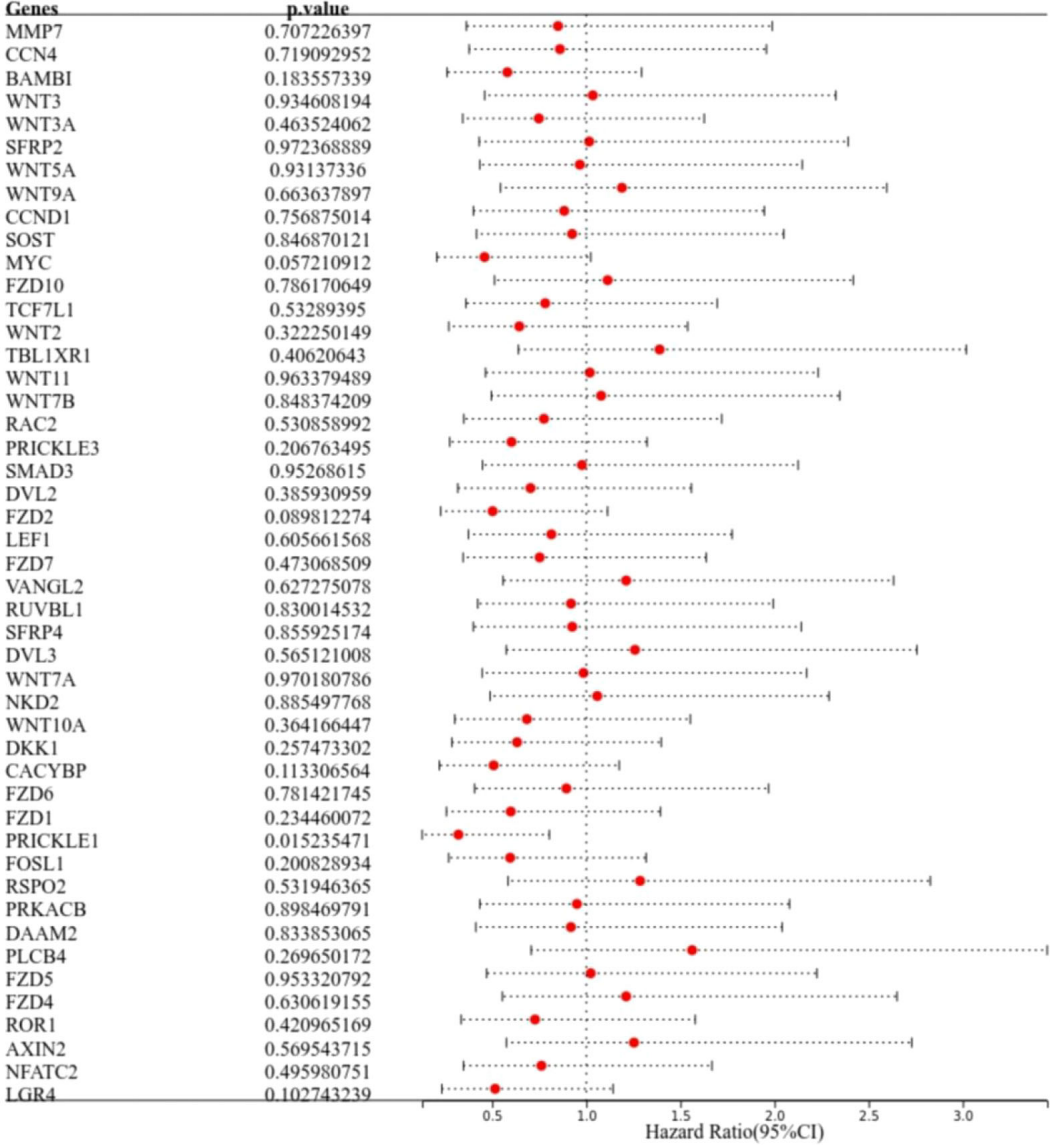
Identification of prognosis-related genes. The forest plot shows PRICKLE1 as a prognosis-related Wnt signaling pathway gene.

The results of one-way COX regression analysis showed that prickle sense protein 1 (PRICKLE1) was a prognosis-related Wnt signaling pathway gene (**Figure 2**, P<0.05).

### 3.3 Nomogram construction

**Figure 3:**
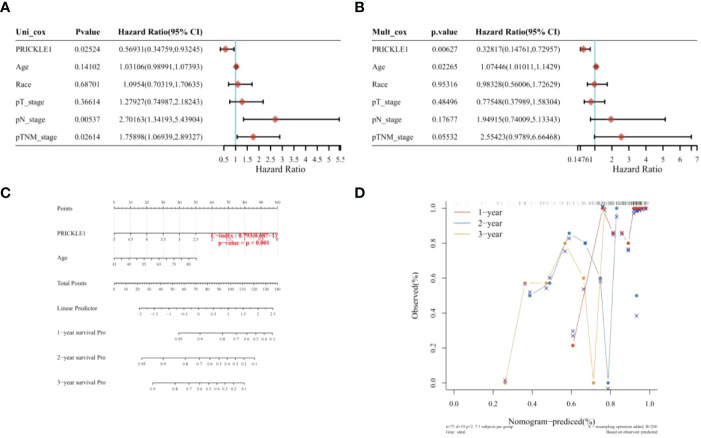
Nomogram construction **(A)** single-factor COX risk regression model; **(B)** multi-factor COX risk regression model; **(C)** column line graph predicting patient survival; **(D)** calibration curve showing the predictive performance of PRICKLE1 on patient survival at 1, 2 and 3 years.

One-way COX risk regression analysis showed that PRICKLE1 was a prognosis-associated Wnt signaling pathway gene (**Figure 3A**, P<0.05). Multi-factor COX risk regression analysis showed that PRICKLE1 may be an independent prognostic factor (**Figure 3B**, P<0.05). Nomogram prediction model showed that PRICKLE1 was an independent prognostic factor in ESCC, and PRICKLE1 had the best predictive ability for a 1-year prognosis in ESCC patients (**Figures 3C, D**).

### 3.4 Single-gene prognostic analysis

**Figure 4:**
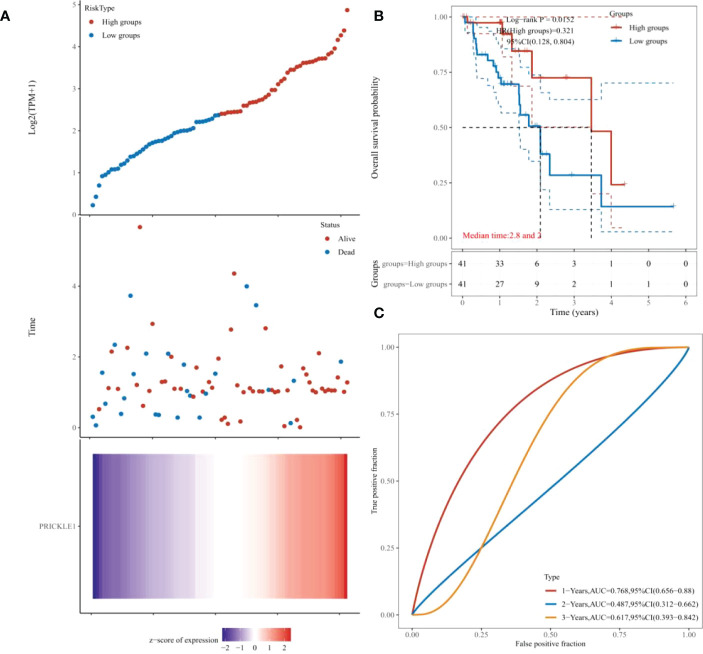
Single-gene prognostic analysis **(A)** Grouping according to individual gene expression; **(B)** Kaplan-Meier survival curves showing patient survival; **(C)** ROC showing the predictive performance of PRICKLE1 on patient survival at 1, 2, and 3 years.

The samples were classified into the PRICKLE1 high expression group and PRICKLE1 low expression group according to the amount of individual gene expression (**Figure 4A**). Kaplan-Meier survival curves showed that the overall survival of patients in the PRICKLE1 high expression group was significantly better (**Figure 4B**, P<0.05). ROC showed that PRICKLE1 predicted 1-year prognosis of ESCC patients with AUC value was the largest (**Figure 4C**).

### 3.5 PRICKLE1 overexpression inhibits cell migration and promotes apoptosis

**Figure 5:**
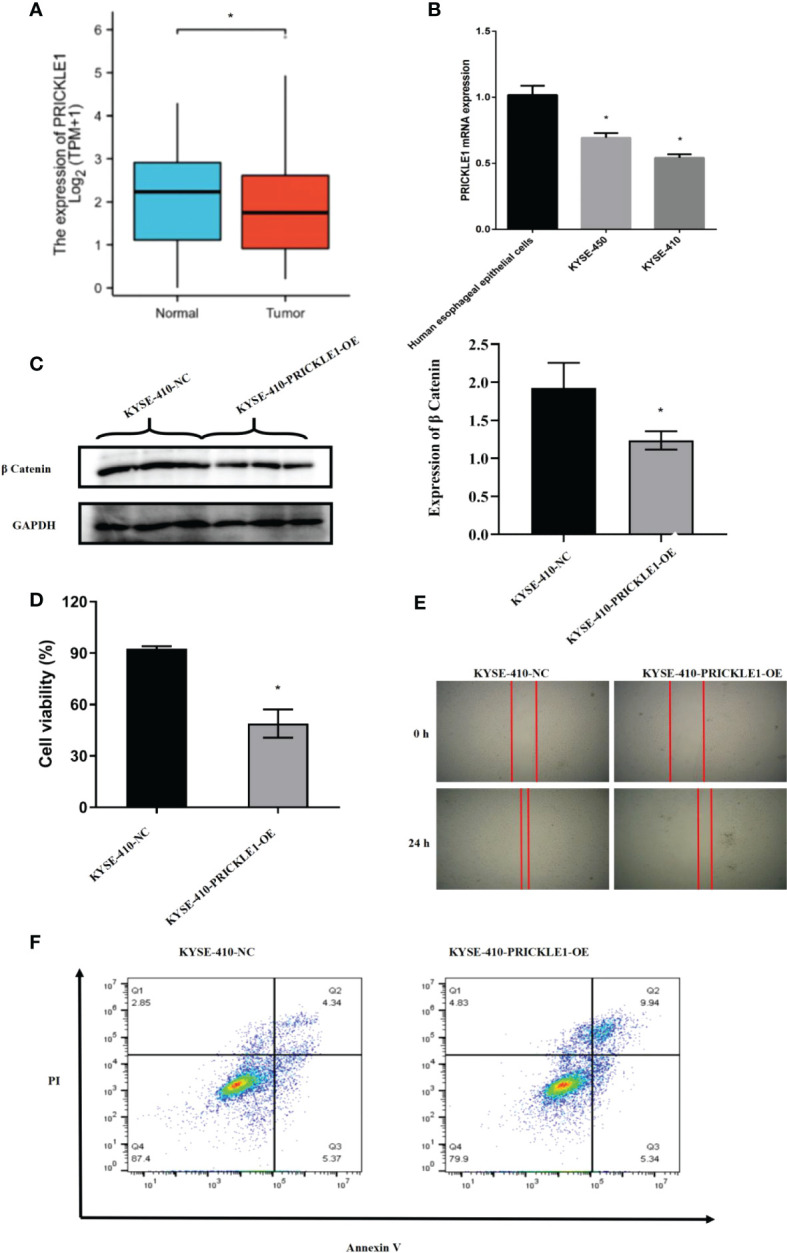
Experimental validation **(A)** Expression of PRICKLE1 in normal human esophageal epithelial cells and esophageal cancer cells; **(B)** qPCR showing mRNA expression in normal human esophageal epithelial cells and esophageal cancer cells; **(C)** WB showing the activation level of Wnt/b-catenin signaling pathway in NC group and PRICKLE1-OE group; **(D)** CCK8 showing cell viability in NC group and PRICKLE1-OE group; **(E)** Cell scratch assay showing cell migration in the NC group and PRICKLE1-OE groups; **(F)** Flow cytometry showing apoptosis in the NC group and PRICKLE1-OE groups, with the sum of cells in Q2 and Q3 in the NC group being 9.71% and the sum of cells in Q2 and Q3 in the PRICKLE1-OE group being 15.28%. Compared with the Human esophageal epithelial cells group, *P<0.05; Compared with the NC group, *P<0.05.

Through experimental validation, we found that the expression of PRICKLE1 was reduced in esophageal cancer; PRICKLE1 was able to attenuate Wnt/β-catenin signaling; the cell viability was reduced, the migration ability was significantly lower and the apoptosis rate was significantly higher in the PRICKLE1-OE group compared with the NC group (**Figures 5A–F**, P<0.05).

## 4 Discussion

The early symptoms of ESCC patients are not obvious and easily ignored by patients and physicians, and patients are often in the middle to late stages when they are clinically diagnosed with ESCC, which is characterized by progressive dysphagia as the main clinical manifestation and a low survival rate (13). Although the techniques for treating ESCC have improved significantly in recent years, the five-year survival rate of the disease is still less than 20% (14). Therefore, there is a clinical need to actively investigate in depth the prognosis-related biomarkers of ESCC to provide important clues and directions for targeted therapy. In recent years, molecular biology has become a hot spot for clinical research, especially the Wnt signaling pathway in tumors (15).

The Wnt signaling pathway is a key pathway in cell development and regulation of growth, which is not only important in early embryonic development, organ formation, and tissue regeneration but also closely related to the cellular carcinogenesis process (16). In a normally growing mature organism, the Wnt signaling pathway is turned off. A mutation in one of the signaling members of the Wnt signaling pathway leads to overactivation of the pathway, resulting in abnormal proliferation and differentiation of many cells, which in turn leads to tumor formation and promotes tumor development (17). It has been shown that abnormalities in the Wnt signaling pathway exist in a variety of human tumors, such as breast, gastric, hepatocellular, colorectal, and prostate cancers (18). The Wnt signaling pathway is a major mechanism in tumor biology, and the activation of this pathway involves various processes such as Wnt2, β-catenin, GSK3β, and Axin. Nakajima M et al. suggested that Axin-2 acts as a negative regulator in the Wnt signaling pathway and is involved in physiological processes such as embryonic development, cell differentiation, and organ formation. Among 81 ESCC patients, five ESCC patients had Axin gene polymorphism as well as reduced Axin protein expression, and low expression of Axin was associated with the clinicopathological characteristics of ESCC patients and was beneficial in predicting the prognosis of patients (19). chu CY’s team concluded that miRNAs related to the Wnt signaling pathway can regulate the development of ESCC cells and affect the prognosis of patients, and Wnt signaling pathway-related genes could be used as potential therapeutic targets for ESCC (20). Deng F’s team found that Wnt2 and β-catenin were highly expressed and GSK3β was lowly expressed in ESCC samples; and the positive rate of Wnt2 was positively correlated with the malignancy of ESCC, the expression of GSK3β was significantly correlated with the tumor location of ESCC, and β-catenin was closely associated with the clinicopathological process in GSK3β-negative ESCC samples (21).

PRICKLE1, a nuclear receptor, is a member of the non-classical Wnt/planar cell polarity (PCP) pathway, where Wnt/PCP signaling controls tissue polarity and cell motility and mediates collective migration events.PRICKLE1 is also a negative regulator in the Wnt signaling pathway, which regulates cells mainly through ubiquitination/deubiquitination and serine/threonine kinase phosphorylation (22).In various cell lines, PRICKLE1 regulates directed cell migration, while at the molecular level, PRICKLE1 regulates the subcellular localization of related proteins as a way to coordinate directed cell migration (23). In different tumor cells, PRICKLE1 has different regulatory roles. On the one hand, it has been found that PRICKLE1 is highly expressed in breast cancer and its upregulation correlates with increased phosphorylation of AKT and its downstream components. PRICKLE1 enhances the migration ability and proliferation of cancer cells by acting on cytoskeleton-related interacting proteins and can be used as a marker of poor prognosis in breast cancer patients (24). Zhou R’s group reported that gastric cancer patients with PRICKLE1 expression were elevated, which may be because PRICKLE1 affects cytoskeletal reorganization by activating mTOR signaling, thus enhancing migration and invasion of gastric cancer cells (25). On the other hand, PRICKLE1 can attenuate Wnt/β-catenin signaling through the degradation inactivation of β-catenin and may act as a tumor suppressor in some cancers (26). It has been suggested that PRICKLE1 is a disheveled (DVL)-associated protein and exerts tumor suppressive effects by antagonizing DVL recruitment; PRICKLE1 expression is reduced in colorectal cancer tissues compared with normal tissues, and PRICKLE1 deficiency may allow further tumor progression (27). In addition, in colorectal cancer patients, PRICKLE1 can directly interact with DVL to mediate ubiquitin-proteasome pathway degradation. Also, PRICKLE1 can negatively regulate Wnt/β-catenin activity, which in turn affects the proliferative activity of colorectal cancer cells (28).

In this study, based on univariate and multifactorial COX risk regression analysis, we observed that PRICKLE1 was an independent prognostic factor in ESCC and that PRICKLE1 had the best predictive power for 1-year prognosis in ESCC patients. In addition, survival analysis showed that patients in the PRICKLE1 high expression group had significantly better overall survival and better prognostic significance in ESCC patients. Through experimental validation, we found that the expression of PRICKLE1 was reduced in esophageal cancer; PRICKLE1 was able to attenuate Wnt/β-catenin signaling; and the cell viability was reduced, the migration ability was significantly reduced and the apoptosis rate was significantly higher in the PRICKLE1-OE group compared with the NC group.This indicates that PRICKLE1 is closely related to the development of ESCC cells. as a class of non-classical Wnt signaling pathway core proteins, PRICKLE1 can effectively inhibit the proliferation and migration of ESCC and increase apoptosis by attenuating the effect of Wnt/β-catenin signaling.

In summary, high PRICKLE1 expression can be used to predict the survival of ESCC patients and can be used as a prognostic indicator for ESCC patients, providing a potential application for ESCC clinical treatment. The present study identified the relationship between PRICKLE1 and the prognosis of ESCC patients, but further elucidation of more detailed regulatory mechanisms is still needed in future studies.In addition, this study has not determined whether Wnt signaling pathway inhibitors are more effective in ESCC patients with high PRICKLE1 expression, and we await further studies in the future.

## Data availability statement

The original contributions presented in the study are included in the article/supplementary material. Further inquiries can be directed to the corresponding authors.

## Author contributions

JH, GL, and HT: writing the article, conception and design, data collection; YW: writing the article, data collection, critical revision of the article; LY: conception and design, analysis and interpretation, data collection; WL: analysis and interpretation, data collection, writing the article; JH: obtaining funding, critical revision of the article, writing the article; WS: critical revision of the article, data collection, writing the article. All authors contributed to the article and approved the submitted version.
